# Deep eutectic solvent-assisted fabrication of bioinspired 3D carbon–calcium phosphate scaffolds for bone tissue engineering†

**DOI:** 10.1039/d3ra02356g

**Published:** 2023-07-20

**Authors:** Marcin Wysokowski, Tomasz Machałowski, Joanna Idaszek, Adrian Chlanda, Jakub Jaroszewicz, Marcin Heljak, Michał Niemczak, Adam Piasecki, Marta Gajewska, Hermann Ehrlich, Wojciech Święszkowski, Teofil Jesionowski

**Affiliations:** a Institute of Chemical Technology and Engineering, Faculty of Chemical Technology, Poznan University of Technology Poznan 60-965 Poland marcin.wysokowski@put.poznan.pl; b Faculty of Materials Science and Engineering, Warsaw University of Technology Warsaw 02-507 Poland; c Lukasiewicz Research Network – Institute of Microelectronics and Photonics, Flake Graphene Research Group 02-668 Warsaw Poland; d Institute of Materials Engineering, Poznan University of Technology Piotrowo 3 61138 Poznan Poland; e Academic Centre for Materials and Nanotechnology, AGH University of Science and Technology Mickiewicza 30 30-059 Kraków Poland; f Center for Advanced Technologies, Adam Mickiewicz University Uniwersytetu Poznanskiego 10 61-614 Poznan Poland

## Abstract

Tissue engineering is a burgeoning field focused on repairing damaged tissues through the combination of bodily cells with highly porous scaffold biomaterials, which serve as templates for tissue regeneration, thus facilitating the growth of new tissue. Carbon materials, constituting an emerging class of superior materials, are currently experiencing remarkable scientific and technological advancements. Consequently, the development of novel 3D carbon-based composite materials has become significant for biomedicine. There is an urgent need for the development of hybrids that will combine the unique bioactivity of ceramics with the performance of carbonaceous materials. Considering these requirements, herein, we propose a straightforward method of producing a 3D carbon-based scaffold that resembles the structural features of spongin, even on the nanometric level of their hierarchical organization. The modification of spongin with calcium phosphate was achieved in a deep eutectic solvent (choline chloride : urea, 1 : 2). The holistic characterization of the scaffolds confirms their remarkable structural features (*i.e.*, porosity, connectivity), along with the biocompatibility of α-tricalcium phosphate (α-TCP), rendering them a promising candidate for stem cell-based tissue-engineering. Culturing human bone marrow mesenchymal stem cells (hMSC) on the surface of the biomimetic scaffold further verifies its growth-facilitating properties, promoting the differentiation of these cells in the osteogenesis direction. ALP activity was significantly higher in osteogenic medium compared to proliferation, indicating the differentiation of hMSC towards osteoblasts. However, no significant difference between C and C–αTCP in the same medium type was observed.

## Abstract 2

Modification of 3D spongin-derived carbon with tricalcium phosphate achieved in a deep eutectic solvent. The 3D carbon–tricalcium phosphate scaffold shows great potential for stem cell-based tissue engineering.

## Graphical abstract

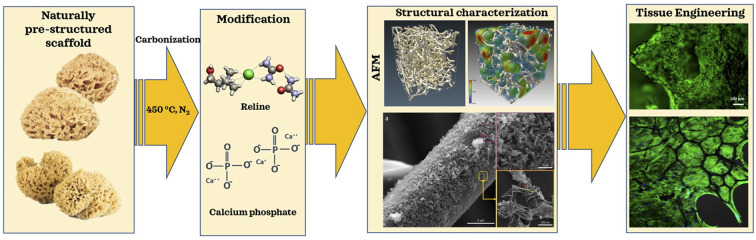

## 1. Introduction

Scaffold creation is often called “*the beating heart*” of regenerative medicine, especially in the tissue engineering (TE) field,^1,2^ due to their crucial role in the creation of an appropriate environment for cell growth, spreading and differentiation. Upon the requirements of combining basic (chemistry, physics, engineering) and life sciences (biology, medicine), this constructive step is still a challenging task.^3^ Typical materials used for the fabrication of tissue engineering scaffolds include synthetic and natural polymers,^4–10^ metals,^11–13^ ceramics,^14,15^ and their combinations. However, a novel approach assumes the creation of advanced bioactive materials that serve as crucial support structures in TE.^16^ These materials offer more effective support for cell growth, and promote the organization of positive physiological responses that expedite the healing process.^16^ In this context, the application of conductive materials with the capability to track cellular behavior in a three-dimensional environment is one of the most fascinating areas of future research. Thus far, several conductive materials have been recognized, *i.e.*, polypyrrole (PPy),^17^ polyaniline (PANI),^18^ and poly(3,4-ethylenedioxythiophene) (PEDOT).^19,20^ However, carbon-based materials, such as graphite,^21^ flake graphene, graphene oxide, carbon nanofibers, and carbon multiwalled nanotubes, offer considerable prospects and are subjected to intensive evaluation for such applications.^22^ Especially, the 3D multiscale carbon-based formations were recently recognized as particularly interesting for bone tissue engineering applications owing to their exceptional mechanical and biological properties.^22–25^ Bone is an electroactive and well-organized structure that spans a range of scales from the nanoscale to the microscale.^16^ Thus, a three-dimensional bioactive carbon-based scaffold seems to be very promising for its regeneration.^24,26–28^ In a study by Dai *et al.*,^29^ a highly porous chitosan/carbon/hydroxyapatite scaffold was established as an agent with enhanced osteoinductivity for bone regeneration. The authors demonstrated that the scaffold materials promoted the growth and differentiation of mouse bone marrow mesenchymal stem cells towards osteogenesis by culturing them on its surface. Additionally, *in vivo* experiments showed that the created composite scaffold significantly enhanced bone formation in the area of an irreparable bone defect.^29^ Special attention was paid to carbon foams as a compromise between the desired spatial structure and material properties. Another study described by Samadian and co-authors^16^ developed osteoconductive and electroactive carbon/hydroxyapatite nanocomposites with properties that were appropriate for bone tissue engineering application. As observed by histomorphometric analysis, the new bone formation was almost 61.3 ± 4.2% higher than that of the negative control. However, the fabrication of highly porous carbonaceous scaffolds with design-controlled geometries provides many difficulties. Formation of 3D open-pore carbon materials is not a trivial task. Most carbons are prepared in the form of powder or flakes, and these materials possess rather unfavorable mechanical properties on the macroscale.^30^ The stiffness of the used materials still does not match the properties offered by their natural counterparts, which have been optimized over time *via* evolution and are appropriately adapted to the performed functions. On the other hand, more sophisticated techniques, such as fused deposition modelling, 3D printing and stereolithography, generate high production costs.^31^

Modern scaffolding strategies based on naturally prefabricated 3D biomaterials also include those of poriferan (sponges) origin.^32^ The skeletons of most marine sponges represent both mineralized^33^ or mineral-free 3D nano-, micro-, and macroporous organic 3D constructs made of chitin,^34^ or proteinaceous spongin.^35,36^ Originally, they have been optimized during the million years of the sponges' evolution with respect to the porosity and mechanical properties for optimal location and functionality of the corresponding cells, which belong to the sponge organism itself. Sponges with both chitin- and spongin-based skeletons show a high regeneration rate under marine farming conditions.^37^ Consequently, scaffolds of such origin have been recognized as renewable biomaterials with high potential for marine bioeconomy,^38^ as well as for TE.^39–41^

Recent findings have shown that carbonization of proteins lead to the formation of carbon that resembles the structure of proteins on all levels of hierarchical organization. We recently reported that by carbonization of spongin (halogenated collagenous structural protein), the carbon sponge not only has the same shape as the original scaffold, but also retains the unique nano-structural characteristics of the collagen triple-helix. As a result, it is mechanically stable and can be cut into any desired shape using a metal saw.^42^ A similar effect has been observed for carbonized silk, wherein the β-sheet structure is converted into a carbon structure that is stacked with polyaromatic units.^43^ The unique hierarchical structure of native sponge skeletons is evolutionarily optimized to ensure proper nutrient diffusion and mechanical stability for cell growth. Semitela *et al.*^44^ used this feature and developed TE scaffolds by coating spongin with graphene.

We propose a straightforward spongin carbonization process. Due to the similar multiple levels of structural hierarchy, we hypothesize that the formed highly porous 3D carbon structures will exhibit specific structural properties that are attractive for cell attachment, growth, and proliferation. Moreover, such carbonaceous 3D structure can be effectively modified with various inorganic nanostructures,^45^*i.e.*, atacamite^46^ or MnO_2_.^47^ Thus, in this study focused on tissue engineering, we decided to modify it with calcium phosphate. Of the variety of materials employed for the regeneration of hard tissue, autograft is the gold standard but there is the limitation of ESI† from the patient.^48,49^ Therefore, calcium phosphate-based materials are used as a synthetic bone graft in the clinical environment, due to their biocompatibility and osteoconduction.^50–59^ These properties allowed its permanent entrance in the history of bone substitute materials.^4,60^ The calcium phosphates, in pure form exhibited not only non-inflammatory, non-toxic^61^ but also osteoconductive properties,^62^ and are able to bond chemically with natural bone tissue.^63,64^ It has been recognized^65^ that diverse metastable calcium-based mineral phases control the evolution of selected scaffold forming proteins. Recently, it has been observed that the incorporation of nano-HAp could improve the cellular activity and viability properties of the porous scaffolds, as well as remarkably stimulate the formation of new bone.^29^ Our unconventional approach is also oriented on the utilization of deep eutectic solvents as a sustainable medium for chemical reactions. Deep eutectic solvents have recently been considered as solvents of the 21st century.^66,67^ They are also confirmed as a useful medium for calcium phosphate formation. Thus, in this study, for the first time, a carbonized spongin scaffold has been covered by calcium phosphate micro- and nanoparticles in a deep eutectic solvent medium. The biomedical potential of the prepared materials has been evaluated by using human mesenchymal stem cells (hMSC) cultured in osteogenic medium for two weeks. The viability was studied qualitatively by live/dead staining and quantitatively using MTS assay; osteogenic differentiation was determined using alkaline phosphatase activity; cell morphology was visualized by means of confocal microscope (cytoskeleton) and scanning electron microscope (cell morphology in context of spongin scaffold).

## 2. Experimental

### 2.1. Preparation of deep eutectic solvent

Deep eutectic solvent – choline chloride : urea (1 : 2) was prepared in a solventless synthesis conducted in a EasyMax 102 (Mettler Toledo, Switzerland) semiautomated reactor system. First, 0.2 mol of choline chloride (Merck, #C1879, purity > 98%, Poland) was mixed with 0.4 mol of urea (Merck, #U5128, purity > 99%, Poland) in a 100 mL reaction vessel at 50 °C for 0.5 h. Subsequently, the obtained mixture was cooled and dried under reduced pressure (0.1 mbar) for 24 h. The product, obtained with a 99% yield, was a colorless liquid containing 8450 ppm of water (assessed with the use of SI Analytics coulometer Titroline 7500 KF Trace). Spectral and physicochemical analysis of choline chloride : urea (1 : 2) has been previously reported by A. P. Abbott *et al.*^68^

### 2.2. Preparation of the 3D carbon–calcium phosphate scaffolds

Prior to carbonization, the spongin scaffold (isolated from *H. communis*, provided by INTIB GmbH, Germany) was treated with 1 M HCl to remove residual CaCO_3_ impurities. The acid solution was replaced with fresh solution every 12 hours. After 48 hours, the scaffolds were washed with distilled water to achieve a neutral pH and dried at room temperature. Then, the obtained precursor was carbonized in an R 50/250/13 tube furnace (Nabertherm, Germany) under a nitrogen atmosphere. The process involved heating the material to 450 °C at a heating rate of 5 °C min^−1^, maintaining it at that temperature for 1 hour, and then allowing it to cool down. Before the carbonization process, the samples were conditioned for 1 hour in a nitrogen atmosphere at a room temperature.

Modification of the obtained scaffolds with calcium phosphate was performed in Easy Max 102 (Mettler-Toledo, Switzerland) adopting and modifying the procedure of calcium phosphate synthesis reported by Karimi *et al.*^69^ In the first step, 0.3 g of CaCl_2_ (Merck, #383147, purity > 96%, Poland) was dissolved in 15 mL of deep eutectic solvent at 100 °C. In the next stage, fragment (1 × 1 × 0.5 cm^3^) of carbonaceous template was placed into the obtained mixture and mixed gently over 30 minutes. After that time, the required amount (Ca/P: 1.67) of 1.62 M aqueous solution of Na_2_HPO_4_ (Merck, #S9763, purity > 99%, Poland) was added with a dosing speed of 0.25 mL s^−1^. The resulting suspension was kept at 100 °C for 1 h and stirred at 200 rpm. Afterwards, the reactor was cooled down to room temperature. The modified scaffolds were isolated using tweezers, and washed with distilled water in an ultrasound bath for 1 h to remove the deep eutectic solvent, as well as the unbounded calcium phosphate nanoparticles.

### 2.3. Characterization of the carbon–calcium phosphate scaffolds

#### 2.3.1. Scanning electron microscopy

The surface and morphology of the obtained 3D carbon–calcium phosphate scaffold was analyzed using a MIRA3 scanning electron microscope (SEM) (Tescan, Czech Republic). The SEM was operated at an acceleration voltage of 2 kV and a working distance of 2 mm. Due to carbon conductivity, the samples were tested without sputtering.

The fixed cell-seeded scaffolds were dehydrated in a series of ethanol dilutions ranging from 70% (v/v) up to absolute ethanol, followed by immersion in hexamethyldisilazane (Fluka, Germany) as a final drying step. Subsequently, the samples were gold-sputtered. The samples after cell cultivation were analyzed using a Phenom ProX microscope (Phenom World, The Netherlands) without any additional preparation, operating at an acceleration voltage of 15 kV in the BSE mode.

#### 2.3.2. Transmission electron microscopy

Transmission electron microscopy (TEM) analysis was conducted using a Tecnai TF20 X-TWIN (FEG) microscope (Thermo Scientific) equipped with an integrated energy dispersive spectrometer (EDAX). The microscope was operated at an accelerating voltage of 200 kV. To prepare the samples for TEM, they were grinded using a mortar, suspended in isopropyl alcohol and drop-casted onto carbon-coated gold TEM grids.

#### 2.3.3. Atomic force microscopy (AFM)

The Bruker Dimension Icon atomic force microscope (AFM) was used to visualize the surface topography and evaluate the stiffness of the bioinspired 3D carbon–calcium phosphate scaffolds. Samples, coated with TCP and non-coated references, were prepared by cutting and attaching them to glass cover slips with carbon tape. The AFM experiments were conducted in air at approximately 21 °C and 33% relative humidity, with the AFM system placed on an anti-vibration table inside an acoustic-proof chamber. Data analysis was performed using Bruker's NanoScope Analysis 1.5 software and the freeware software Gwyddion (version 2.56).

For topographical examination, a silica ACT 50 scanning probe with a nominal radius below 10 nm was employed. Prior to imaging, the scanning probe frequency was estimated at around 278 kHz using the Auto-Tune function in the Nanoscope software. Tapping Mode was used to visualize the scaffold surface, focusing exclusively on individual fibers to avoid artifacts from curvature. A scanning area of 1 × 1 μm^2^ was selected to capture subtle topographical features, and the acquired data were analyzed to evaluate surface roughness.

To assess mechanical stability, the OTESPA-R3 scanning probe by Bruker was mounted into the AFM. The setup was calibrated with known mechanical properties using calibration samples. Surface stiffness in the nanoscale was examined using Quantitative NanoMechanics Mode (QNM). Mechanical testing was limited to the top of the fibers, and stiffness maps with a resolution of 256 × 256 points were generated. The Derjaguin-Muller-Toporov (DMT) contact mechanic model was employed to fit the recorded experimental data.

Overall, the Dimension Icon AFM facilitated visualization of the surface topography and provided insights into the stiffness properties of the 3D carbon–calcium phosphate scaffolds.

#### 2.3.4. Micro computer tomography (μ-CT)

The specimens' fragment, which measured approximately 3 × 4 × 4 mm^3^, was subjected to *μ-CT* image acquisition. The imaging was carried out using an X-ray tomographic system (MICRO XCT-400, Xradia-Zeiss, Pleasanton, CA, USA) with the following settings: 40 kV voltage, 10 W power, no filter material, 0.16° rotation step in an angle interval of 184°. The voxel size was 5 × 5 × 5 μm^3^. Subsequently, Avizo Fire (Thermo Fisher Scientific, USA) was utilized for the data processing, image analysis and 3D reconstruction of the scanned samples.

### 2.4. Biological evaluation

#### 2.4.1. Human mesenchymal stem cells (hMSC) culture

Human bone marrow mesenchymal stem cells (hMSC) used in this study were purchased from Lonza (USA). The cells were cultured in the Minimum Essential Medium alpha (α-MEM, Gibco, UK) supplemented with 10% fetal bovine serum (FBS, EuroClone, Italy), 1% penicillin and streptomycin (PS, Gibco, USA) and 1 ng mL^−1^ human basic fibroblast growth factor 2 (FGF2, Sigma-Aldrich, Israel).

#### 2.4.2. hMSC seeding procedure

The spongin scaffolds were sterilized by soaking them in 70% ethanol for 1 h, after which samples were rinsed three times with autoclaved deionized water, followed by a single wash with PBS. Next, the scaffolds were allowed to incubate overnight in expansion medium. Samples were seeded with 2 × 10^5^ cells per sample. On the next day, the samples were transferred to new wells containing either expansion medium (control for ALP activity assay, see Section 2.5.4) or osteogenic medium, *i.e.*, α-MEM supplemented with 10% FBS, 1% PS 10 mM β-glycerophosphate (Sigma, USA), 50 μg mL^−1^l-ascorbic acid 2-phosphate (Sigma, Japan), 10 nM of vitamin 1α, 25-dihydroxy-vitamin D3 (Sigma, Israel) and 10 nM of dexamethasone (Sigma, China). The medium was replaced every 2 days.

#### 2.4.3. Live–dead cells staining

At predetermined time points, the samples were washed with DPBS (Gibco, UK) and incubated with live–dead fluorescent dyes: acridine orange (Sigma, 3 μg mL^−1^, green fluorescence, all cells) and propidium iodide (Sigma, 10 μg mL^−1^, red fluorescence, dead cells) dissolved in PBS for approximately at 37 °C for 5 min. Afterwards, the constructs were washed with DPBS and examined using a fluorescence microscope (Leica TCS SP8) at wavelengths corresponding to fluorophores of interest (green fluorescence – viable cells, red fluorescence – dead cells).

#### 2.4.4. Cytoskeleton staining

The cell-seeded scaffolds were fixed using 4% (w/v) paraformaldehyde solution in PBS for 15 min, and permeabilized by means of 0.2% v/v Triton X-100/PBS for 10 min. After incubation in 1% v/v BSA solution for 15 min, the samples were immersed in a Phalloidin conjugated with Alexa Flour 488 (1 : 40 ratio v/v, in 0.1% BSA/PBS solution) for 2 h. Afterwards, samples were rinsed with PBS and nuclei were counterstained with Draq5 (1 : 1000 ratio v/v, Thermo Scientific, USA) for 15 min. The stained samples were analyzed using a confocal microscope (Leica TCS SP8, day 1 and day 7) and (Zeiss Axio Observer 7, day 14) at excitation wavelengths of 488 nm and 633 nm.

#### 2.4.5. Cell viability: MTS assay

Cell viability was quantified after 14 days of culture using the CellTiter 96® AQueous One Solution Cell Proliferation Assay (Promega, USA). After a wash with α-MEM w/o FBS, the samples were moved to a new 24-well plate containing 1 mL of α-MEM w/o FBS and 200 μL of MTS, and incubated for 90 min. The supernatant was transferred in quadruplicate to a 96-well plate (100 μL per well) and the absorbance was measured using a microplate reader (Fluostar Omega, BMGLabtech, Germany) at *λ* of 490 nm. To determine the cell number, hMSC were seeded at densities ranging from 0 to 1 × 10^5^ cells per well and after overnight incubation, the MTS assay was performed in the same manner. The absorbance was then plotted against the known cell number and used to calculate the equation describing the standard curve.

#### 2.4.6. Cell differentiation: alkaline phosphatase (ALP) activity

To determine the ALP activity, the cell-seeded samples were washed with DPBS, moved into new wells containing 1 mL of *para*-nitrophenyl phosphate (pNPP) solution (Phosphate Substrate Kit, Thermo Scientific), and incubated at room temperature for 45 min. An aliquot containing 100 μL of the solution was subsequently transferred in quadruplicate to wells of 96-well plate, and the absorbance was measured at *λ* of 405 nm. The results were normalized to the total DNA content measured using the cell lysate and CyQuant™ Proliferation assay kit (Thermo Fisher, USA), following the protocol delivered by the manufacturer. The cell lysate was obtained by freezing the samples in 1 mL of demineralized H_2_O, followed by 3 thawing–freezing cycles.

### 2.5. Statistical analysis

The data, which were presented as a mean ± standard deviation (SD), were analyzed using post hoc one-way ANOVA with a Tukey–Kramer pair-wise comparison test (KyPlot 2.0 beta 15 software) to determine statistical significance.

## 3. Results and discussion

**Fig. 1:**
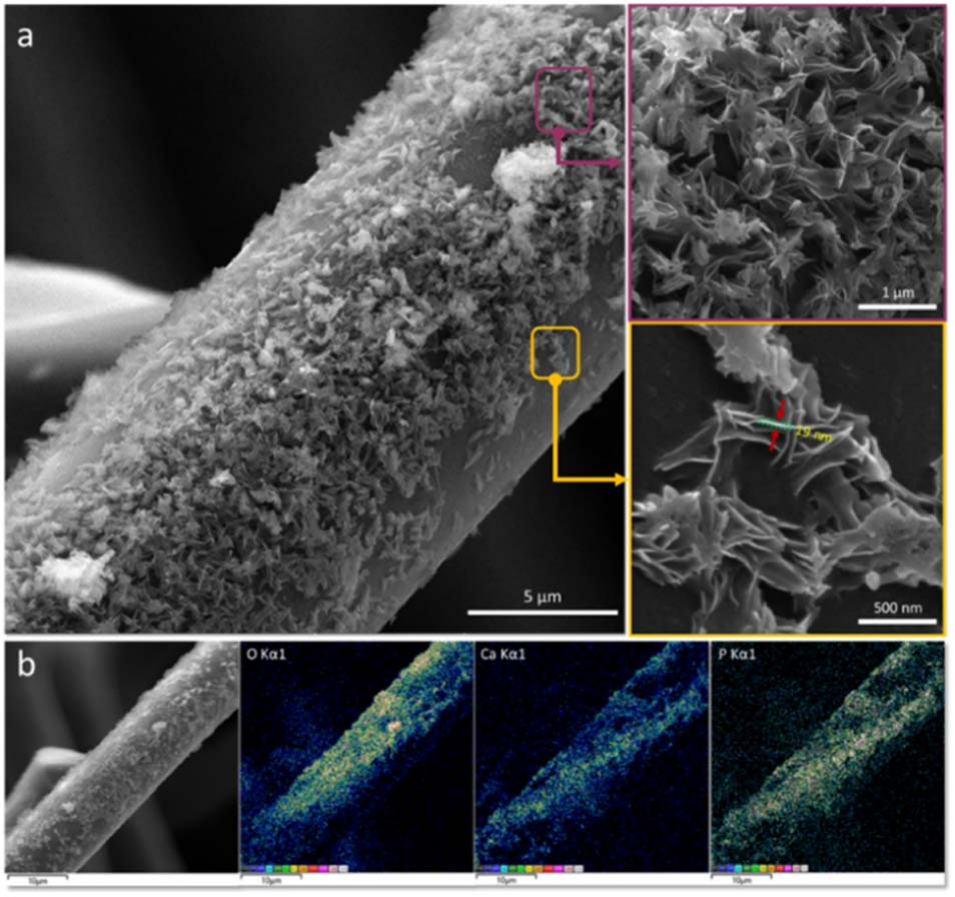
(a) Scanning electron microscopy images, with different magnitudes, of carbonized spongin covered with inorganic nanoparticles. (b) EDX mapping reflecting their elemental composition with respect to calcium, phosphorus and oxygen.

The surface morphology of the carbonized spongin (as reference) and carbonized spongin coated with inorganic nanoparticles is depicted in Fig. S1† and 1a, respectively. The SEM images presented in Fig. 1a show that the nanoparticles that are homogenously deposited on the surface of the carbonized spongin fibers are characterized with uniform plate-like morphology. The thickness of these nanoparticles oscillates around 19 nm, and these nanoparticles are tightly affixed to the surface of the spongin-derived carbonaceous template. These nanostructures could not be detached from the surface even after subjecting them to 1 h of ultrasound treatment.

The EDX mapping (Fig. 1b) confirms that the nanoparticles are composed of calcium and phosphate. The Ca : P ratio of the deposited nanoparticles is equal to 1.5 (Fig. S2, ESI†).

**Fig. 2:**
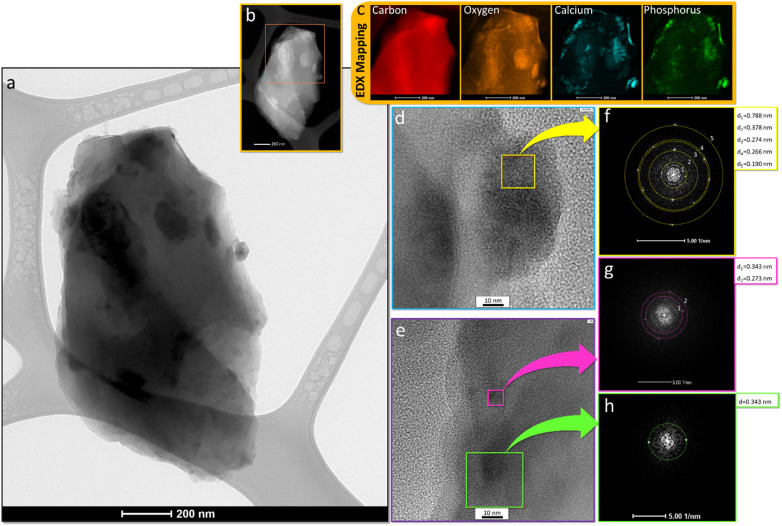
(a) Overview bright-field and (b) dark-field TEM images recorded for carbonized spongin covered with calcium phosphate nanoparticles. (c) EDX mapping of the entire region of the analyzed composite. (d and e) HR-TEM images of the selected region of the composite with corresponding (f–h) FFT analysis from the local regions.

Fig. 2 shows the TEM images in light (Fig. 2a) and dark mode (Fig. 2b) of the nanocomposite prepared in this study, together with the elemental mapping (Fig. 2c). The turbostratic graphite that is formed by carbonization of spongin is confirmed using high-resolution transmission (Fig. 2d and e) electron microscopy (HRTEM) and fast Fourier transformation^42^ (Fig. 2). The Fourier transform (Fig. 2f) displays the diffraction maxima of 0.788 nm^−1^ corresponding to a spacing of 25.6 Å and originating from the diffraction plane (002).^70–72^

The registered diffraction maxima of 0.378 nm, 0.274 nm, 0.266 nm and 0.190 nm (Fig. 2f–h) correspond with the planes (202), (−231)/(−132), (411)/(114), and (−623)/(404) of the monoclinic Ca_3_PO_4_, respectively (PDF 04-018-9895).

**Fig. 3:**
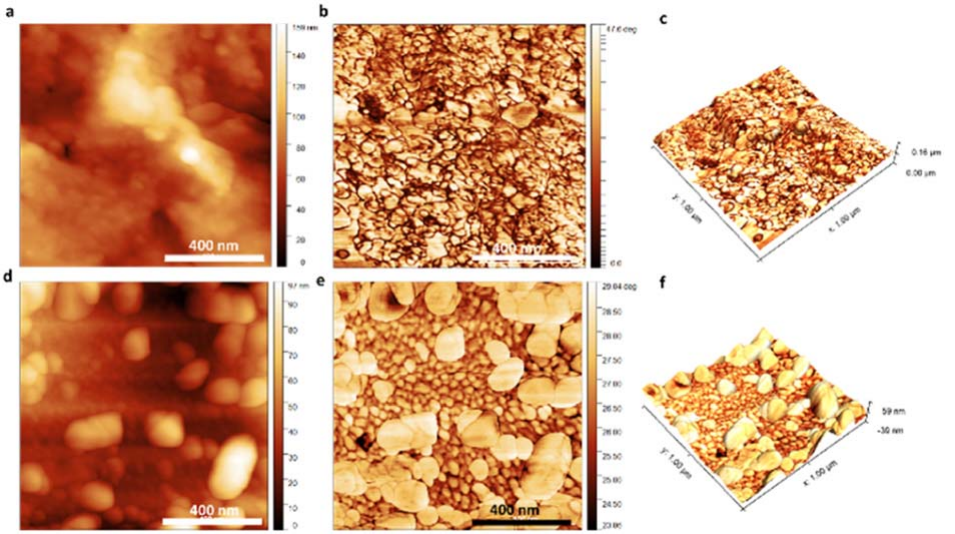
AFM visualization of the surface of the 3D scaffolds: (a) and (d) depict the topography of the reference and αTCP-covered scaffold, (b) and (e) depict the phase-contrast images of the reference and αTCP-covered scaffold, and (c) and (f) depict 3D topography reconstruction with overlapped PC data of the reference and αTCP-covered scaffold.

To scrutinize the subtle topographical variations of the 3D carbon–TCP scaffolds in the nanoscale, we utilized the AFM technique working in Tapping Mode. Given the fibrous, openwork architecture and the inherent curvature of individual fibers of the tested scaffolds, we decided to visualize only small areas on the top of the fibers. This enabled us to record high quality topographical and phase-contrast (PC) images of the surface of the scaffolds (Fig. 3).

The acquired images were used for both qualitative and quantitative analysis of the surface – average roughness (*R*_a_), and the root mean square roughness (RMS) parameters were calculated based on the topographical maps. From the data summarized in Table 1, it can be perceived that the surface of the pristine carbonous samples was smoother in comparison with the surface of the scaffolds covered with αTCP.

**Table 1:** Surface features quantified based on the AFM topographical maps

Parameter	Pure carbonous sample	Carbon–αTCP
Roughness (*R*_a_) (nm)	3.48 ± 0.49	9.54 ± 2.42
Roughness (RMS) (nm)	4.76 ± 0.90	12.46 ± 2.73

This conclusion was further justified by the AFM-image based topographical assessment of the surface of the tested samples.

A correlation was found between the ceramic particles on the surface of the individual fibers and the surface's presence. Bright areas on both topographical (meaning that the particles were higher than the carbonous matrix) and phase-contrast (meaning that the particles were characterized with different properties than the carbonous matrix) images registered in the case of the carbon–αTCP scaffolds should be associated exclusively with the presence of the αTCP particles. In particular, the 3D topographical images with the overlapped PC maps depicted the carbonous fibers encrusted with αTCP particles, thus bolstering the roughness of the surface.

**Fig. 4:**
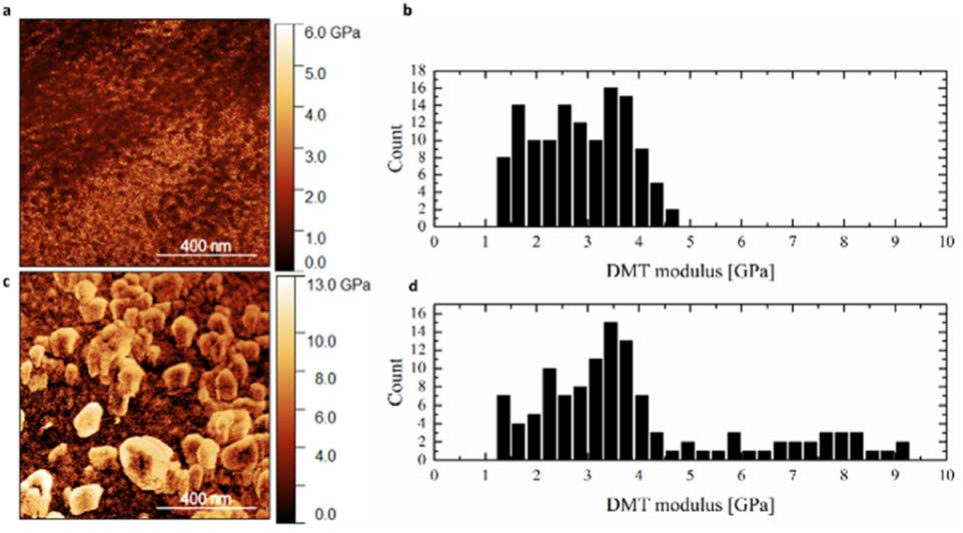
AFM QNM stiffness maps of the (a) reference and (c) αTCP covered scaffolds, followed by DMT modulus histograms (b) and (d).

AFM operating in Quantitative NanoMechanics mode was implemented to estimate the stiffness of the surface of individual fibers of the scaffolds. Fig. 4 illustrates the representative AFM QNM stiffness maps and stiffness histograms calculated based on the DMT contact mechanic's model. It can be observed that the pristine carbonous fibers were characterized with rather uniform mechanical properties (Fig. 4a and b). It is worth noting that the QNM maps of the surface of the samples covered with calcium phosphate nanoparticles looked different in comparison with the QNM maps of the reference samples. We were able to detect a stiffer phase, whose presence was manifested by distinct bright areas (Fig. 4c). This phenomenon could originate from the αTCP presence on the surface of the tested fibers. It should be underlined that this stiffer phase altered the distribution of the registered stiffness values (Fig. 4d) in comparison with the stiffness histogram obtained for the reference samples (Fig. 4b). Apart from the main contribution (narrower part of the histogram at 1.4–4.5 GPa) coming from the carbonous matrix of the fibers, an additional share from the stiffer phase (wider part of the histogram 4.5–9.3 GPa) was registered. Altogether, the topographical and mechanical studies in the nanoscale performed with AFM manifested that the surface of the fibers was successfully covered with αTCP particles. Moreover, the ceramic particles enhanced roughness of the fibers and simultaneously fortified the mechanical properties of their surface.

The mechanical properties of the investigated sponges were identified. In Fig. S3,† the stress–strain curves obtained in the monotonic compressive test are presented. Generally, three stages could be distinguished during the compression of the porous/spongious structures. The first one is the linear elastic stage, where the structure follows Hook's law. In the second stage, the cell/pore walls start to buckle, which manifests as the characteristic stress plateau. In the third stage, the densification of the spongious structure takes place, *i.e.*, the porous spaces collapse completely,^73^ and a bulk block is formed. During the densification stage, a notable stress increase is observed. No clear evidence of failure could be detected from the presented curves. However, it is relatively easy to distinguish the two subsequent compression stages. The first of the observed stages is the plateau region, whereas the linear stage is somewhat absent or very narrow. One can see that the stress notably increased when the sponges were compressed to more than 60% of strain, which may indicate the densification stage presence. The compressive moduli of the reference and CHAP groups determined in the plateau region of the stress–strain curve were 0.48 ± 0.08 MPa and 0.54 ± 0.03 MPa, respectively.

**Fig. 5:**
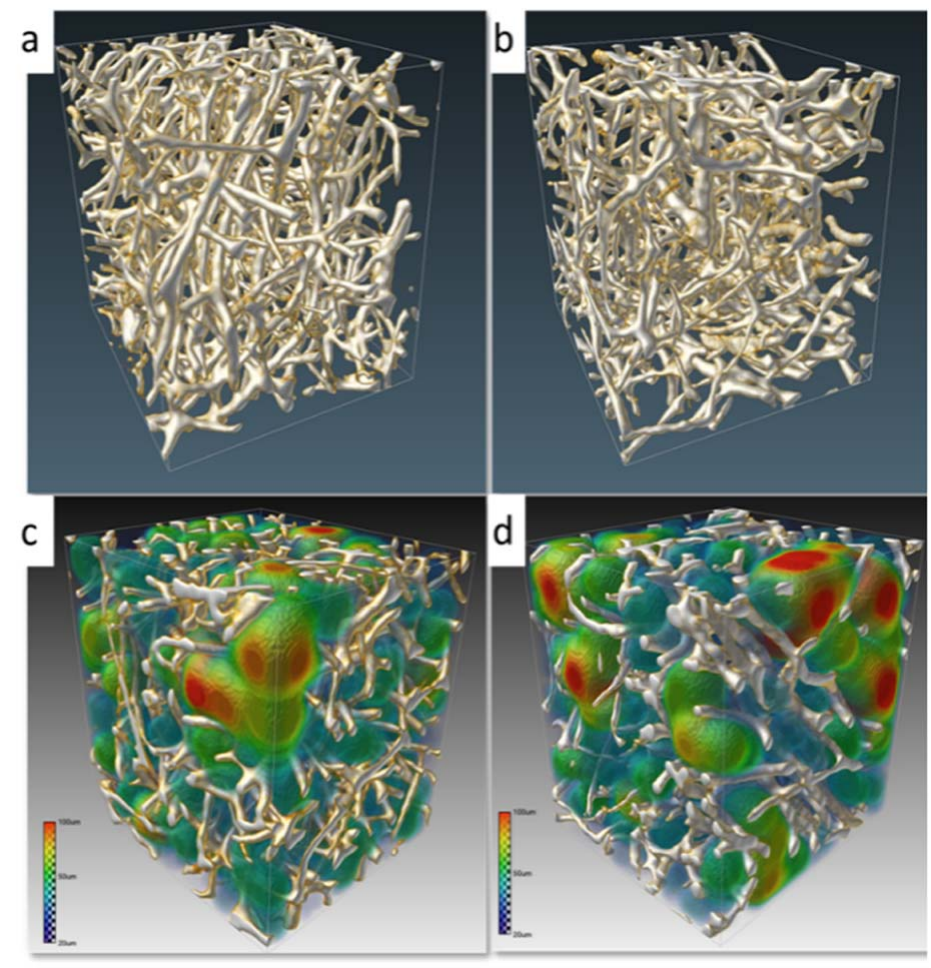
Three-dimensional visualization obtained from μCT analysis of the carbonized spongin (a) scaffold before and (b) after covering by αTCP. (c) and (d) Colored maps of the pore size distribution of the reference and modified sample, respectively.

Quantification of the level of porosity, connectivity, and material density of the obtained scaffolds by micro-CT is presented in Fig. 5a–d and Table 2. Apart from providing information about the porosity of the scaffolds, the 3D evaluation of micro-CT additionally provides an assessment of the connectivity or interconnection of the pores. The obtained results indicate that an applied modification does not have a negative impact on the scaffold porosity, which is 87%.

**Table 2:** Quantitative data on the porosity, scaffold fiber thickness distributions and structure separation, obtained by analysis of the μCT data

Parameter	Pure carbonous sample	Carbon–αTCP
Porosity (%)	86.5 ± 0.39	87 ± 0.43
Structure thickness [μm]	12.6 ± 2.7	12.6 ± 2.8
Structure separation [μm]	48.5 ± 18.7	49.3 ± 22.5

The colored maps of the pore size distribution clearly demonstrate that the modified scaffold is composed of interconnected open pores that are essential for nutrient flow and scaffold colonization, and for bone ingrowth (osteoconduction) associated with both angiogenesis and osteogenesis.^74^

It is reported that the ideal scaffold requires a porosity between 60% and 90%.^75^ The obtained results confirm our hypothesis that the sponge-derived scaffolds are perfectly optimized toward cell and tissue support. This also highlights their unique potential as naturally prefabricated, structured constructs for tissue engineering.^38,76^

### 3.1. Cell viability

**Fig. 6:**
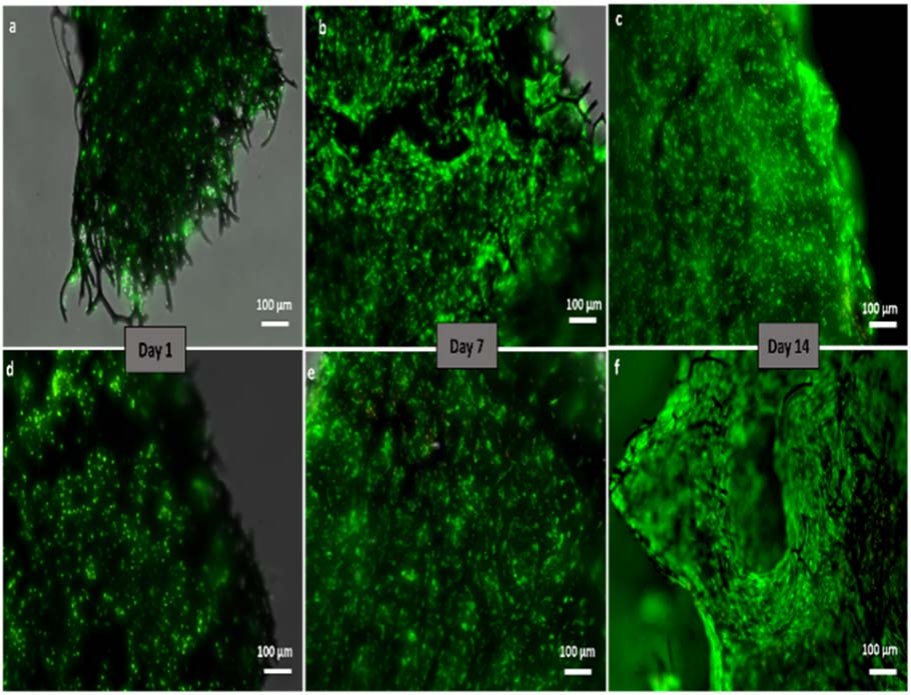
Fluorescence microscope images represent the results of live–dead staining of hMSC cells seeded on (a–c) carbonized spongin and (d–f) carbon–TCP scaffolds. Live cells (green), dead cells (red).

Qualitative analysis of hMSC viability was performed by means of live/dead staining. As can be seen in Fig. 6, there was virtually no red fluorescence and a simultaneous abundance of green fluorescence at each time-point, *i.e.*, after 1, 7 and 14 days of culture.

Moreover, the intensity of green fluorescence increased during the culture, indicating hMSC proliferation. This allows us to conclude that the spongin-derived carbon scaffolds, both pristine and tricalcium phosphate-coated, were not cytotoxic and supported hMSC growth.

### 3.2. Cell morphology

**Fig. 7:**
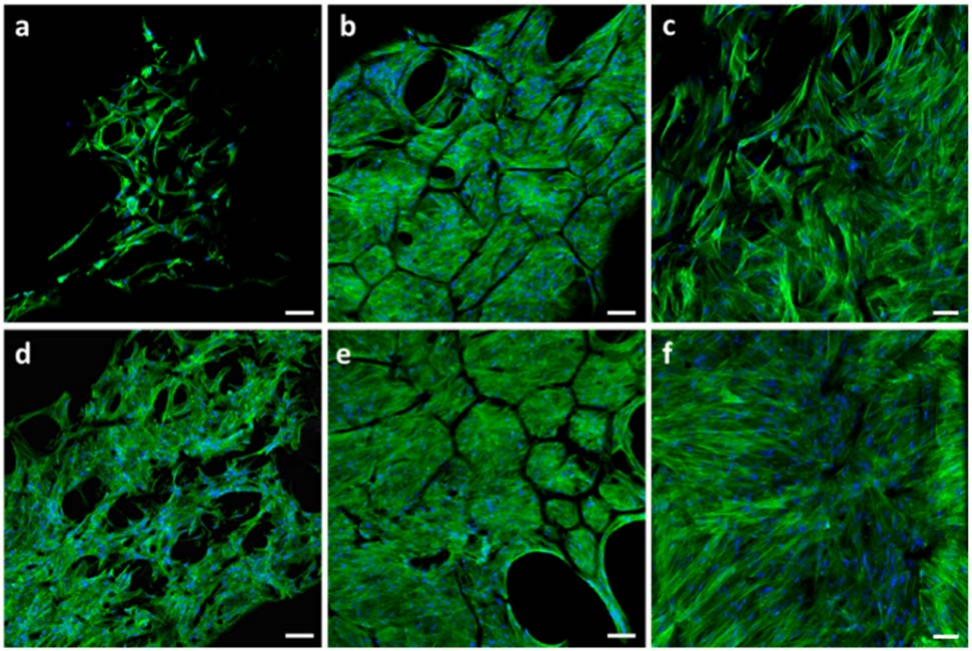
Confocal microscope images depicting the morphology and organization of the cytoskeleton of hMSC seeded on (a–c) pristine and (d–f) TCP-coated carbonized spongin scaffolds (C–αTCP). Images were taken after 1 (a and d), 7 (b and e) and 14 days (c and f) after seeding. The cells were stained for F-actin (green) and nucleus (blue). Scale bar: 100 μm (a, b, d and e) and 50 μm (c and f).

The hMSC adhered well to both tested materials since day 1 of culture. The cells were spread along the spongin, resembling carbonaceous struts at lower cell density, and bridged or even closed the struts at higher cell density (see Fig. 7a and d). The F-actin microfilaments were visible and aligned along the axis of the cells. Upon culture, the cell density increased significantly, and the hMSC started filling and closing the gaps between the struts.

Following the first week of culture (Fig. 7b and e), it was virtually impossible to distinguish one cell from another. Therefore, it is difficult to discuss the cell morphology based on the observations using confocal microscopy.

**Fig. 8:**
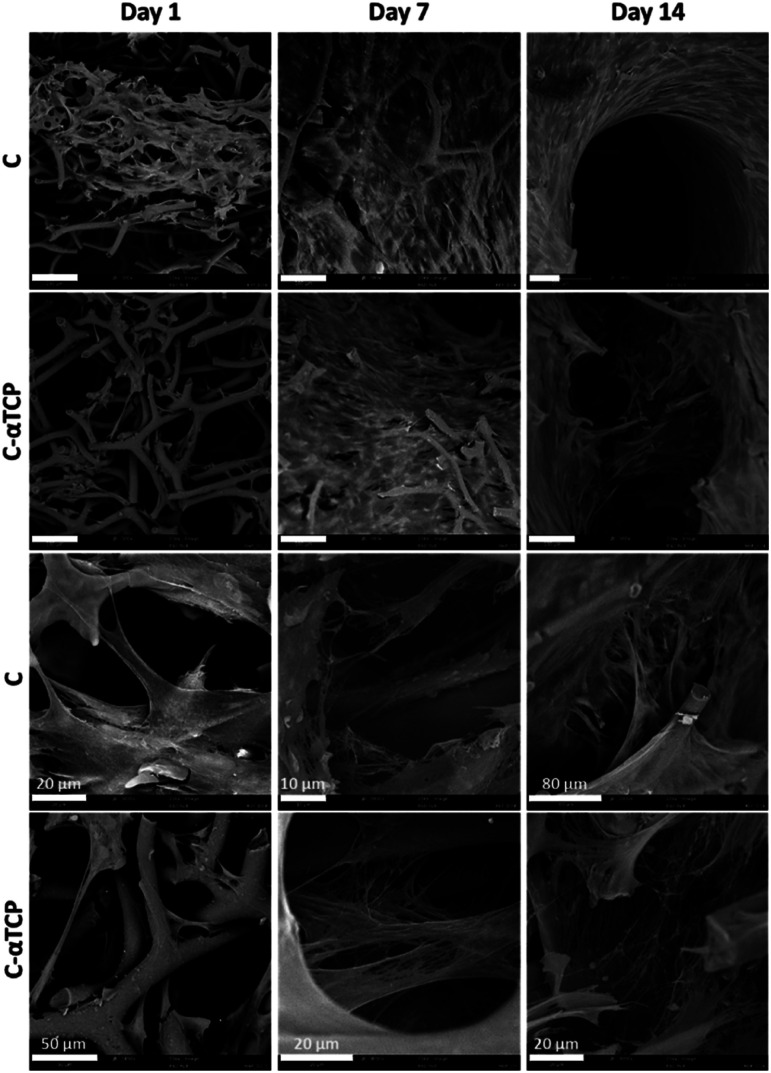
SEM observations of hMSC growing on a carbon scaffold (C) and carbon–α tricalcium phosphate (C–αTCP) scaffolds over 2 weeks of culture. Scale bar = 100 μm (unless indicates otherwise). The top two rows depict the overall distribution of the cells, whereas the two bottom rows depict the cell morphology and network of ECM.

SEM observations (Fig. 8) revealed that one day post-seeding, the hMSC were attached to the scaffolds. Moreover, they were extending filopodia to the bridge struts of the scaffolds. The filopodia were longer in the areas of lower cell density and they were generally extending along the struts. However, in some instances, they were wrapping the struts. HMSC already formed a dense cell sheet after 1 week of culture. Currently, the point, fibrillar network of the extracellular matrix (ECM) was visible under the cell sheet. The density of both cell sheet and the ECM network increased over the next week of culture. Secretion of the fibrillar ECM network composed predominantly of collagen type I is considered as an early marker of osteogenic differentiation of MSC, and was found to further foster the osteogenic differentiation.^77,78^

### 3.3. Quantitative analysis of the hMSC viability and differentiation

**Fig. 9:**
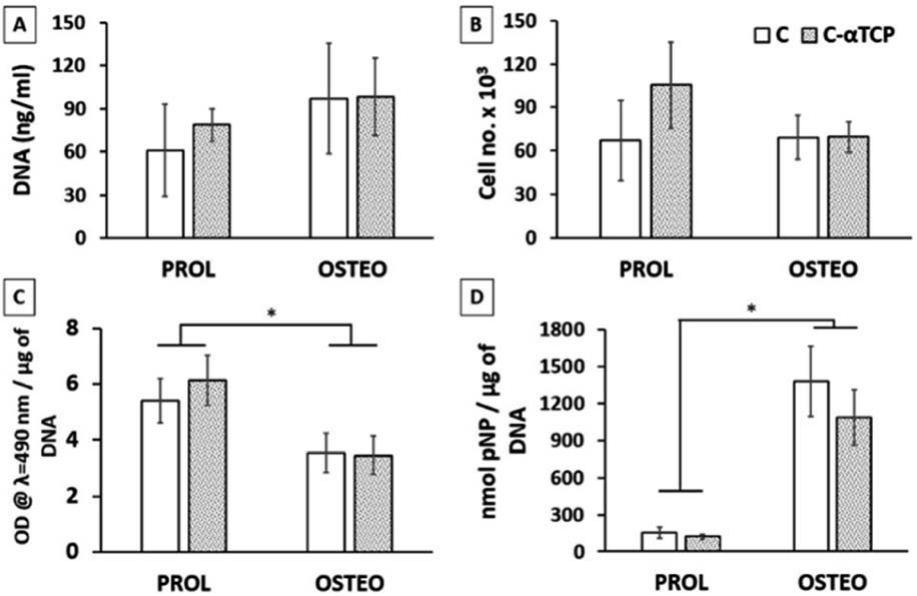
Quantitative characterization of hMSC viability and differentiation after 14 days of culture: (a) concentration of DNA in the tested samples; (b) cell number determined using the MTS assay; (c) cell metabolic activity measured using the MTS assay normalized to the DNA content; (d) ALP activity normalized to the DNA content (*p < 0.05).

After two weeks of culture, we did not measure any significant difference in the DNA content and cell number determined by the MTS assays on the two tested materials in either proliferation or expansion medium (Fig. 9a and b). According to the MTS assay, the number of viable cells was in the range of 70–100 × 10^3^ per scaffold. However, the standard curve describing the correlation between the cell number and metabolic activity was prepared by culturing the cells in a monolayer. Therefore, the real cell number might vary due to different behaviors of cells cultured in 3D-fashion.^79^ Interestingly, we observed significant differences in the viability of cells cultured in the proliferation and differentiation media when the absorbance measured by means of the MTS assay was normalized to the total DNA content (Fig. 9c). This could be a result of the higher metabolic activity of hMSC in the proliferative state than upon differentiation on the investigated materials. Blahnova and co-workers observed a similar trend when culturing hMSC in osteogenic medium supplemented with growth factors enhancing osteogenic differentiation.^80^

The ALP activity was significantly higher in the osteogenic medium compared to the proliferation medium (Fig. 9d), indicating differentiation of hMSC towards osteoblasts. However, we did not see any significant difference between C and C–αTCP in the same medium type. Those results suggest that the content of αTCP was too low to influence the osteogenic differentiation of hMSC. Indeed, studies investigating the effect of ions that could be released from αTCP upon dissolution, *e.g.*, Ca^2+^, showed that concentrations above 4 mM enhanced the osteogenic differentiation of hMSC.^81,82^

## 4. Conclusions

The findings presented and discussed in this study showcase a novel method involving a deep eutectic solvent for the creation of novel biomimetic nanostructured carbon–tricalcium phosphate composites. The developed approach allowed for the efficient and homogenous deposition of uniform TCP nanoparticles on the spongin-derived carbonaceous template. This highlights the unique potential of deep eutectic solvents as a sustainable medium in the creation of hybrid materials for advanced applications.

Spongin-derived carbon 3D-scaffolds are superior to other templates due to their renewable nature, straightforward preparation process, and intricate architecture. The extraordinary structural features (open porous fiber architecture), along with the biocompatibility and mechanical properties of α-TCP, render them as an excellent candidate for diverse biomedical applications, particularly in stem cell-based tissue-engineering. Ultimately, future biomimetic research should be oriented toward the replication and prototyping of spongin structural organization by means of high-resolution additive manufacturing with the use of other high-performance materials and supported by machine learning techniques to optimize and tune it precisely for final application. Such holistic approach will be definitively ground-breaking in terms of biomimetic materials science.

## Author contributions

Marcin Wysokowski: writing original draft, supervising, resources, investigation, founding acquisition, visualization; Tomasz Machałowski: writing original draft, investigation, visualization; Joanna Idaszek: writing original draft, investigation, visualization; Adrian Chlanda: writing original draft, investigation, visualization; Jakub Jaroszewicz: investigation, visualization; Marcin Heljak: writing original draft, investigation, visualization; Michał Niemczak: resources, writing – review and editing; Marta Gajewska: investigation, visualization; Adam Piasecki: investigation, visualization; Hermann Ehrlich: writing – review and editing, resources; Wojciech Święszkowski: writing – review and editing, resources; Teofil Jesionowski: writing – review and editing, resources.

## Conflicts of interest

There are no conflicts to declare.

## Supplementary Material

RA-013-D3RA02356G-s001
