# Supplementary material for: Deep eutectic solvent-assisted fabrication of bioinspired 3D carbon–calcium phosphate scaffolds for bone tissue engineering
Source: RSC Adv. 2023 Jul 20;13(32):21971–81. doi: 10.1039/d3ra02356g (PMC10358318; doi:10.1039/d3ra02356g)
Supplement: RA-013-D3RA02356G-s001 [file RA-013-D3RA02356G-s001.pdf]

Electronic Supplementary Material (ESI) for RSC Advances.
This journal is © The Royal Society of Chemistry 2023

Electronic supplementary information:

Deep eutectic solvent-assisted fabrication of bioinspired 3D carbon-calcium phosphate scaffolds for bone tissue engineering

Marcin Wysokowski,[a]* Tomasz Machałowski,[a] Joanna Idaszek,[b] Adrian Chlanda,[c] Jakub Jaroszewicz,[b] Marcin Heljak,[b] Michał Niemczak,[a] Adam Piasecki,[d] Marta Gajewska,[e] Hermann Ehrlich,[f,g] Wojciech Święszkowski,[b] Teofil Jesionowski[a]

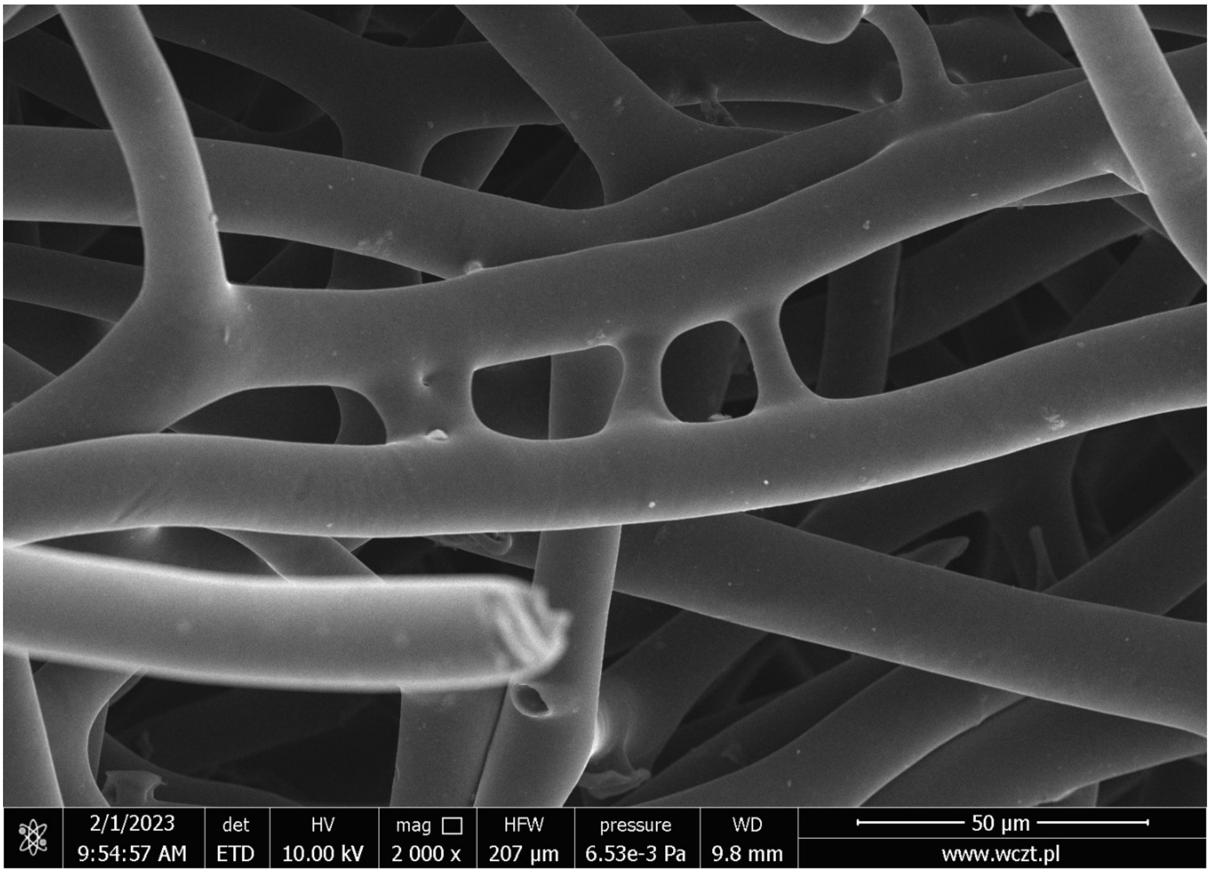

Figure S1. Scanning electron microscopy image of reference carbonized spongin.

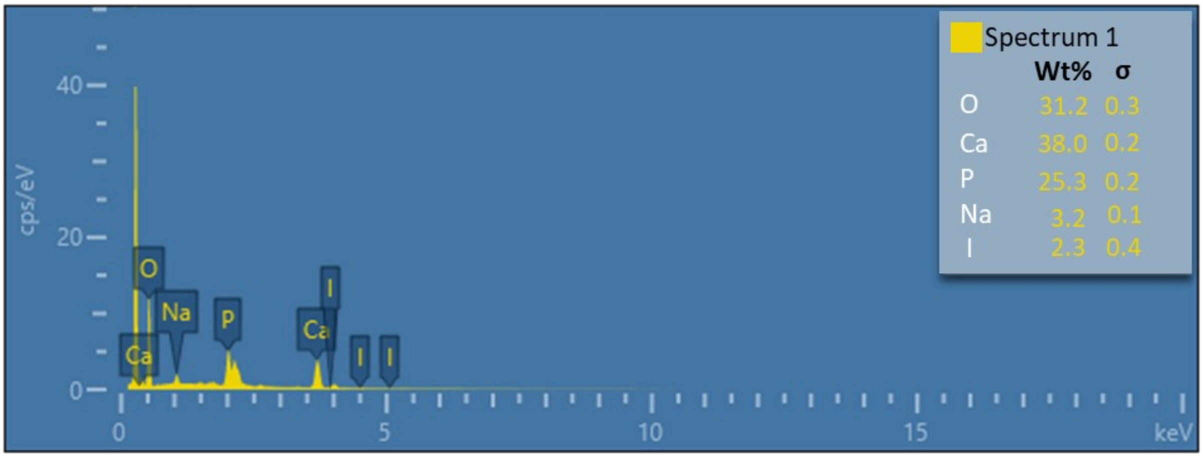

Figure S2. EDX analysis of elemental composition of deposited nanoparticles.

The monotonic compression test was carried out aiming for the quantifying compressive properties of the specimens. The test was carried out using the Q800 (TA Instruments, USA) Dynamic Mechanical Analysis (DMA) instrument. All the specimens were tested in the air at room temperature. Specimens were prepared with a square prism shape of width ca. 1 mm and thickness ca. 1 mm. In the case of the monotonic test, all specimens were preloaded to 0.01 N and compressed at a constant strain rate of 5%/minute. Compressive moduli as the ratio between compressive stress and strain in the linear portion of the stress-strain curve (at 20 % of total strain) were identified.

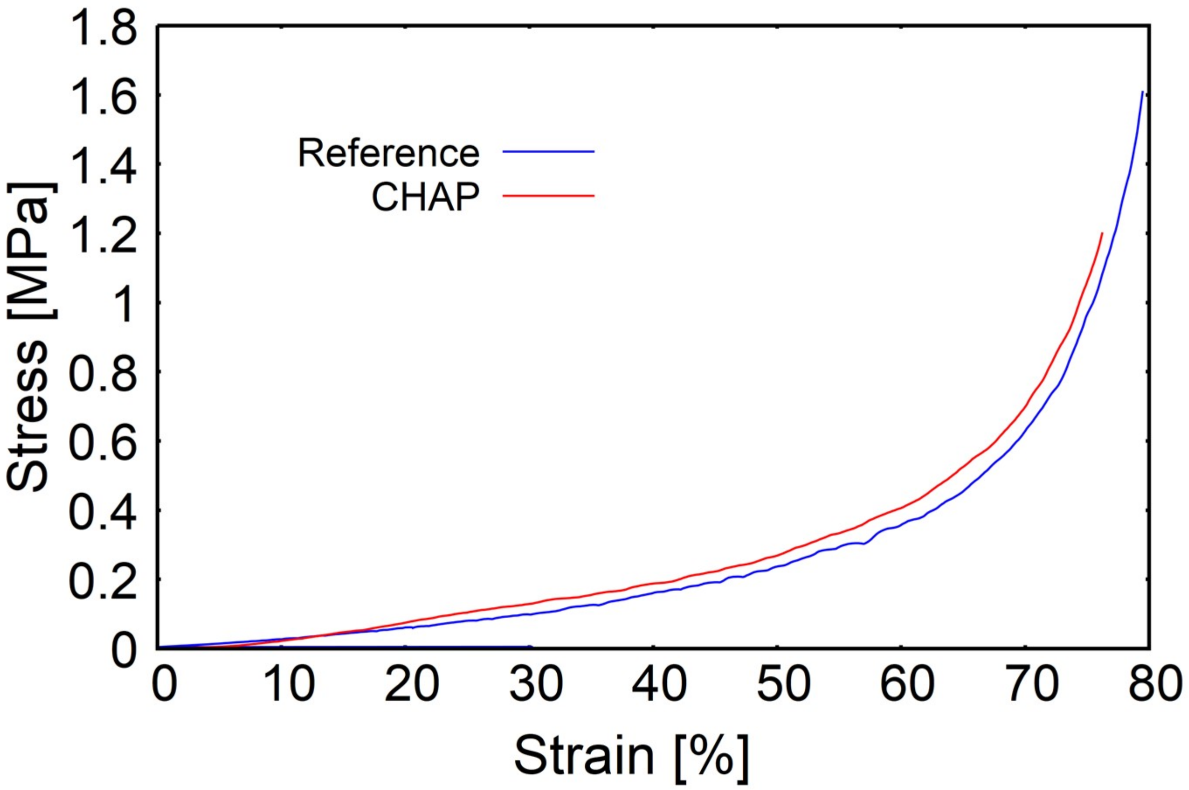

Figure S3. Strain-stress response of investigated spongious structures; (mean ± SD; *n* = 5)
